# Effects of a new medical insurance payment system for hospice patients in palliative care programs in Korea

**DOI:** 10.1186/s12904-018-0300-x

**Published:** 2018-03-07

**Authors:** Youngin Lee, Seung Hun Lee, Yun Jin Kim, Sang Yeoup Lee, Jeong Gyu Lee, Dong Wook Jeong, Yu Hyeon Yi, Young Jin Tak, Hye Rim Hwang, Mieun Gwon

**Affiliations:** 1 Department of Family Medicine, Pusan National University Hospital, Busan, 602-739 South Korea; 2 Biomedical Research Institute, Pusan National University Hospital, Busan, 602-739 South Korea; 3 Medical Education Unit and Medical Research Institute, Pusan National University School of Medicine, Yangsan, 626-870 South Korea; 4 Obesity, Nutrition and Metabolism Clinic, Department of Family Medicine and Research Institute of Convergence of Biomedical Science and Technology, Pusan National University Yangsan Hospital, Yangsan, 626-770 South Korea; 5 Pusan National University Yangsan Hospital, Yangsan, Gyeongsangnam-do 626-770 South Korea; 6 Department of Family Medicine and Biomedical Research Institute, Pusan National University Hospital, Busan, 49241 South Korea

**Keywords:** Hospice, Palliative, Insurance, Payment system, Length of survival

## Abstract

**Background:**

This study investigates the effects of a new medical insurance payment system for hospice patients in palliative care programs and analyzes length of survival (LoS) determinants.

**Method:**

At the Pusan National University Hospital hospice center, between January 2015 and April 2016, 276 patients were hospitalized with several diagnosed types of terminal stage cancer. This study separated patients into two groups, “old” and “new,” by admission date, considering the new system has been applied from July 15, 2015. The study subsequently compared LoS, total cost, and out-of-pocket expenses for the two groups.

**Results:**

Overall, 142 patients applied to the new medical insurance payment system group, while the old medical insurance payment system included 134 patients. The results do not show a significantly negative difference in LoS for the new system group (*p* = 0.054). Total cost is higher within the new group (*p* <  0.001); however, the new system registers lower patient out-of-pocket expenses (*p* <  0.001).

**Conclusion:**

The novelty of this study is proving that the new medical insurance payment system is not inferior to the classic one in terms of LoS. The total cost of the new system increased due to a multidisciplinary approach toward palliative care. However, out-of-pocket expenses for patients overall decreased, easing their financial burden.

## Background

Cancer is one of the most lethal diseases worldwide, with the highest mortality rate, which further increases in advanced stages [1]. When the cancer cannot be controlled anymore, a healthcare team determines whether to stop medical testing and treatment [2]. For such patients, medical facilities can provide palliative care, which includes not only medical treatment to alleviate pain and symptoms, but also psychological, social, and spiritual therapies [2, 3]. The purpose of palliative care is providing benefit during survival and improving the quality of life for patients and their families [2, 4–6]. As such, the need for these services is increasing [7, 8].

However, the existing Korean national medical insurance system is a fee-for-service system that covers only medical treatment, thus making it impossible for patients and their families to obtain any psychological, social, or spiritual support via the national insurance system [9]. Moreover, it also does not cover admission room and individual nursing fees [9]. As a result, the economic burden for these services is a well-known social issue [9, 10]. For these reasons, the Ministry of Health and Welfare of Korea adopted a new insurance system from July 15, 2015, based on diagnosis-related groups (DRG) and per diem payment to support patients through a multidisciplinary approach and lighten their economic burden [9]. The DRG payment system was previously adopted in seven surgical groups, such as appendectomy and cesarean delivery [11]. There are several studies that evaluated the effect of the DRG system in surgical groups [12, 13] or other countries [14–16], but also on the effect of other payment systems in palliative care [17, 18]. For the new payment system for palliative care in Korea, only the length of stay and cost analyses were performed during the demonstration period [19]. However, there is no research on medical effects, such as length of survival (LoS), for the new payment system in palliative care after its adoption. The goal of this study is to investigate the effect of the new payment system in hospice programs.

## Methods

This study analyzes data from the Pusan National University Hospital (PNUH) hospice center and retrospectively studies patients receiving palliative care for cancer. The study population is represented by patients hospitalized between January 1, 2015 and April 25, 2016 in the PNUH hospice center. These patients are registered into the palliative care program, which provides only supportive care, not curable treatments. We obtained data on LoS, defined as the length of time from first admission to death. Our secondary variable, cost, is separated into four categories. Total cost refers to the total amount of hospitalized cost, including insurance coverage and out-of-pocket expenses for patients. We also divided total cost and out-of-pocket expenses for patients by hospitalized period and analyzed baseline characters (sex, age, religion, primary cancer type, liver metastasis, bone metastasis, type of treatment received [chemotherapy, radiotherapy, surgery], pain grade, Eastern Cooperative Oncology Group(ECOG) score), laboratory data determinants for the survival period (white blood cell, hemoglobin, segmented neutrophil, lymphocyte, platelet, aspartate aminotransferase, alanine aminotransferase, alkaline phosphatase, lactate dehydrogenase, total bilirubin, albumin, blood urea nitrogen, creatinine, sodium, potassium, c-reactive protein) [20–22].

We separated the patients into two groups, “old payment system group” and “new payment system group,” by admission date, divided as of July 15, 2015, when the new system was applied. Patients hospitalized before and discharged after July 15, 2015 were treated as censored on July 15, 2015 in the LoS analysis and excluded from the cost analysis, because we cannot separate cost data according to the introduction date of the new system. We statistically analyzed length of survival by the Kaplan–Meier curve and log rank test method. We also performed Cox proportional hazard analysis to investigate the effect of the payment system on LoS with the variables on baseline characteristics and laboratory findings. Regarding cost analysis, we compared the average total cost and out-of-pocket expenses for patients by the Mann–Whitney test. Additionally, non-parametric rank analysis of covariance (ANCOVA) was used to adjust for pain grade.

These analyses were performed with R 3.2.0, using R studio. We considered statistical significance for *p*-values below 0.05.

## Results

### Participating population

We enrolled 276 adult patients with advanced cancer, who received palliative care in the PNUH hospice center between January 1, 2015 and April 25, 2016. The characteristics of the study participants are presented in Table 1. From these, 134 patients belong to the old payment system group and 142 to the new payment system group. Of the 134 patients in the old payment system, the number of males is 71 (53%) and the median age is 68. In the other group, 80 (56.3%) are male and the median age is also 68. Only two variables show a statistical difference in baseline characteristics between groups: the pain grade, which is higher in the new system group (6.00 [4.00, 6.00]) than the old system group (3.00 [2.00, 4.00]), and albumin, which is higher in the old system group (3.30 g/dL) than in the new system group (3.10 g/dL). There were no significant differences in other variables.

**Table 1:** Baseline characteristics

	Old system	New system	*p*-value
n	134	142	
Sex = M (%)	71 (53.0)	80 (56.3)	0.661
Age	68.00 [58.00, 74.00]	68.00 [59.25, 74.75]	0.759
Admission date (days)	16.00 [9.00, 35.00]	15.50 [6.00, 29.75]	0.217
ECOG score(%)			0.075
0	1 (0.7)	0 (0.0)	
1	7 (5.2)	3 (2.1)	
2	33 (24.6)	49 (34.5)	
3	77 (57.5)	65 (45.8)	
4	16 (11.9)	25 (17.6)	
Pain grade^a^	3.00 [2.00, 4.00]	6.00 [4.00, 6.00]	< 0.001
Religion (%)			0.650
None	55 (41.0)	53 (37.3)	
Buddhism	56 (41.8)	56 (39.4)	
Christian	16 (11.9)	22 (15.5)	
Catholic	7 (5.2)	11 (7.7)	
Primary cancer site (%)			0.659
Colon cancer	16 (11.9)	24 (16.9)	
Stomach cancer	17 (12.7)	13 (9.2)	
Pancreatic cancer	9 (6.7)	13 (9.2)	
Hepato-cellular carcinoma	4 (3.0)	5 (3.5)	
Lung cancer	18 (13.4)	22 (15.5)	
Cervix cancer	3 (2.2)	1 (0.7)	
Breast cancer	14 (10.4)	9 (6.3)	
Prostate cancer	2 (1.5)	4 (2.8)	
Other malignancy	51 (38.1)	51 (35.9)	
Liver metastasis = 1 (%)	12 (9.0)	24 (16.9)	0.075
Bone metastasis = 1 (%)	18 (13.4)	18 (12.7)	0.994
Chemotherapy = 1 (%)	91 (67.9)	94 (66.2)	0.861
Radiotherapy = 1 (%)	43 (32.1)	42 (29.6)	0.748
Surgery = 1 (%)	69 (51.5)	67 (47.2)	0.552
Laboratory results^b^			
White blood cell (× 10^3^/uL)	9.31 [6.71, 13.58]	9.68 [7.09, 13.64]	0.606
Hemoglobin (g/dL)	10.70 [9.30, 12.10]	10.10 [9.00, 11.40]	0.160
Segmented neutrophil (%)	76.70 [68.30, 84.50]	78.10 [70.85, 85.10]	0.310
Lymphocytes (%)	13.60 [7.90, 19.90]	11.40 [6.45, 17.35]	0.154
Platelet (×10^3^/uL)	243.00 [163.00, 303.00]	232.00 [151.00, 320.50]	0.586
Aspartate aminotransferase (IU/L)	27.00 [17.25, 48.00]	27.00 [19.00, 67.50]	0.370
Alanine aminotransferase (IU/L)	15.00 [10.00, 28.75]	16.00 [10.50, 34.00]	0.411
Alkaline phosphatase (IU/L)	118.00 [69.25, 249.50]	141.00 [78.50, 297.50]	0.417
Lactate dehydrogenase (IU/L)	284.00 [189.00, 422.00]	316.00 [228.50, 492.00]	0.061
Total bilirubin (mg/dL)	0.60 [0.38, 0.90]	0.67 [0.40, 1.71]	0.217
Albumin (g/dL)	3.30 [2.90, 3.60]	3.10 [2.80, 3.50]	0.036
Blood urea nitrogen (mg/dL)	18.70 [14.50, 28.40]	20.50 [13.35, 30.45]	0.991
Creatinine (mg/dL)	0.80 [0.63, 1.13]	0.80 [0.61, 1.20]	0.719
Sodium (mmol/L)	135.15 [131.97, 137.48]	134.20 [130.60, 137.30]	0.377
Potassium (mmol/L)	4.38 [3.91, 4.74]	4.21 [3.84, 4.58]	0.121
C-reactive protein (mg/dL)	4.30 [1.40, 9.85]	5.61 [2.05, 11.99]	0.145

^a^Pain grade is from 1 (lowest) to 10 (highest) and is determined at admission

^b^Laboratory results presented as median values (95% confidence interval)

### Length of survival

**Fig. 1:**
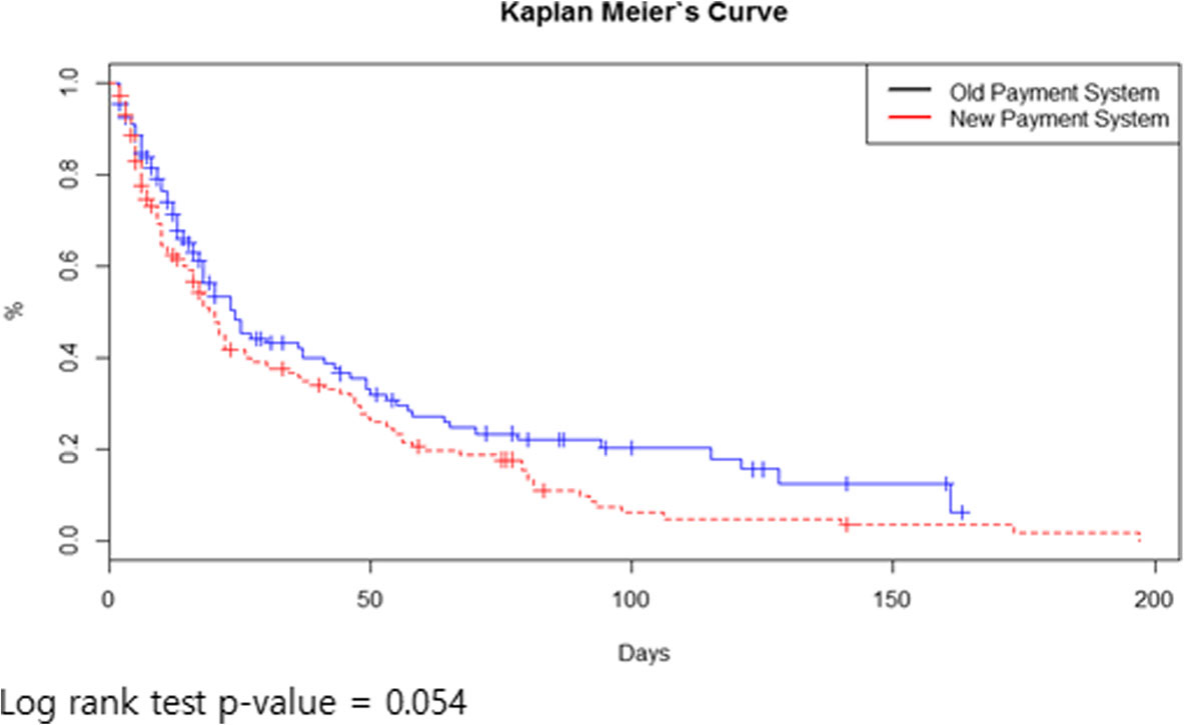
Kaplan Meier’s survival curve (log rank test *p*-value = 0.054)

In Fig. 1, the old payment system group has a slightly higher LoS curve than the new one, but its log rank test result has a *p*-value of 0.054, which is not statistically significant. In Table 2, both groups have the same median LoS of 16 days. There is slightly higher hazard ratio (1.31 [0.99, 1.73]) when performing Cox proportional analysis, but it is not statistically significant, having a *p*-value of 0.055. There is also no significant hazard ratio (1.24 [0.80, 1.93]), with a *p*-value of 0.334 when we adjusted all variables.

**Table 2:** Length of survival analysis

Type of payment system	Cases	Length of survival (days)^a^	Crude model		Adjusted model	
			HR	*p*-value	HR	*p*-value
Old payment system	134	16 (8.00, 43.75)	Reference	Reference	Reference	Reference
New payment system	142	16 (6.00, 45.50)	1.31 (0.99, 1.73)	0.055	1.24 (0.80, 1.93)	0.334

^a^The results are presented as median values

### Cost

**Fig. 2:**
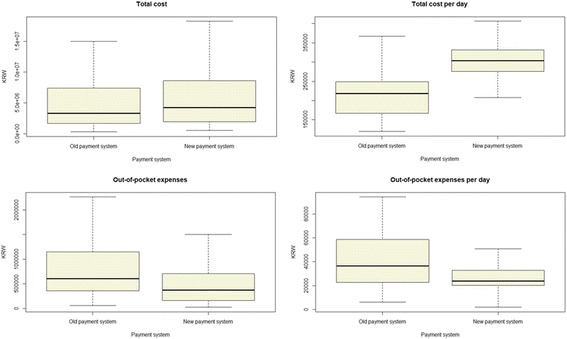
Cost analysis

In Table 3 and Fig. 2, during the admission period, total medical cost was higher in the new payment system (KRW 4244000) compared to the old one (KRW 3313000), and was not statistically significant (*p* = 0.071). However, out-of-pocket expenses for patients were significantly lower (*p* < 0.001) in the new payment system (KRW 375000) than the old (KRW 603000). We also compared per diem total medical cost and out-of-pocket expenses for patients. In the new payment system, total cost per diem was higher (KRW 304000 > 218,000), and out-of-pocket expenses for patients per diem lower (KRW 24000 < 37,000), with a significant *p*-value (*p* < 0.001). After adjusting for pain grade, there was significant difference in per diem cost (F = 108.366, *p* < 0.001) and out-of-pocket expenses (F = 15.218, *p* < 0.001).

**Table 3:** Cost analysis

	Old system	New system	*p*-value^a^	Adjusted *p*-value^b^
Case #	134	142		
Total cost	3,313,000 (1,713,000, 7,338,000)	4,244,000 (1,964,000, 8,551,000)	0.071	
Out-of-pocket expenses	603,000 (358,000, 1,146,000)	375,000 (166,000, 704,000)	< 0.001	
Cost/Day	218,000 (166,000, 248,000)	304,000 (276,000, 331,000)	< 0.001	< 0.001
Out-of-pocket expenses/Day	37,000 (23,000, 59,000)	24,000 (20,000, 33,000)	< 0.001	< 0.001

KRW is used as the cost unit. The results are presented as median values

^a^*P*-values are calculated using the Mann–Whitney U-test

^b^Adjusted *p*-values were calculated using a rank ANCOVA adjusted for pain grade

## Discussion

In this study, we evaluated the effect of a new payment system based on DRG and per diem cost in palliative care. As a primary result, there was no significant difference in LoS with the adjustment of various factors. Another research point is represented by the economic issues. As previously mentioned, the goal of the new payment system is decreasing patients’ economic burden. Per our results, total budget increased, but out-of-pocket expenses for patients decreased in the new payment system.

Previous studies presented LoS as an index to evaluate patients’ quality of life [23]. Our study proved that the new payment system is not inferior in terms of LoS. Therefore, the new payment system will not negatively affect patients’ quality of life.

The role of a multidisciplinary team is important in relieving the overall pain of cancer patients in palliative care [24]. However, proper rewards were not distributed to the team in the old system. Moreover, due to the limitations of the old system, multidisciplinary support was not fully provided to hospice patients [9]. Total cost had to increase because the new payment system supports multidisciplinary approaches, including psychological, social, and spiritual consultancy. As such, the new payment system guarantees adequate payment for social workers in multiple departments. Therefore, patients and their families could be provided various services to improve quality of life. Moreover, the government covers a broad spectrum of hospice services by the new payment system. Consequently, the out-of-pocket expenses for patients decreased, that is, their economic burden, compared to the old system. Economic issues are one of the biggest concerns for both patients and their caregivers, and reducing economic stress can also improve quality of life [3, 25]. Further, we expect the hospice program to become more widespread and assist more cancer patients and their caregivers.

To the best of our knowledge, this is the first study on the effects of the new payment system with hospice care in Korea, which was adopted in July 2015. Given data collection and analysis time, our results are timely.

However, this study has certain limitations. First, it is a single center study, conducted in PNUH, Pusan, Korea, and there could be a selection bias due to hospital characteristics. Second, the limited target population could also be an issue. However, a single center study was preferred because there could be differences in grade, capable range of intervention therapies, and nursing payment methods by hospital, which can affect cost analysis. Second, we cannot evaluate patients’ subjective satisfaction, which is important in determining quality of life [3, 26, 27]. The study plan was drawn after the new payment system has been adopted, making it impossible to administer questionnaire surveys targeting patients within the old payment system. This could also be a disadvantage of the retrospective study method. Third, there is significant difference in pain grade at the admission date, which can possibly cause higher analgesic costs for the group reporting a higher pain grade and can also be associated with higher medical costs. Although there is statistically significant difference in cost and out-of-pocket expenses after adjusting for pain grade, well-controlled research would be needed. Finally, our study only evaluated hospitalized patients registered into a palliative care program. Due to the program criteria, we did not include patients who received palliative care in outpatient clinics or concurrently with a curative treatment.

For further research, analysis including multiple centers would be needed to clarify the relationships between cost, LoS, and quality of life.

## Conclusions

This study evaluated the effect of the new payment system for hospice programs. It was found that LoS was not statistically different between selected groups. Total cost increased in the new payment system group without statistical significance, but out-of-pocket expenses for patients decreased within the same group with statistical significance. The results meet the purpose of the new system.

## Abbreviations

DRG: Diagnosis-related groups
ECOG: Eastern Cooperative Oncology Group
LoS: Length of survival
PNUH: Pusan National University Hospital
